# Manipulation-induced hypoalgesia in musculoskeletal pain populations: a systematic critical review and meta-analysis

**DOI:** 10.1186/s12998-018-0226-7

**Published:** 2019-01-29

**Authors:** Sasha L. Aspinall, Charlotte Leboeuf-Yde, Sarah J. Etherington, Bruce F. Walker

**Affiliations:** 1 School of Health Professions, Murdoch University, Perth, WA Australia; 2 Institute of Regional Health Research, University of Southern Denmark, Odense, Denmark; 3 School of Veterinary and Life Sciences, Murdoch University, Perth, WA Australia

**Keywords:** Spinal manipulative therapy, Quantitative sensory testing, Pain sensitivity, Hypoalgesia, Musculoskeletal pain

## Abstract

**Background:**

Manipulation-induced hypoalgesia (MIH) represents reduced pain sensitivity following joint manipulation, and has been documented in various populations. It is unknown, however, whether MIH following high-velocity low-amplitude spinal manipulative therapy is a specific and clinically relevant treatment effect.

**Methods:**

This systematic critical review with meta-analysis investigated changes in quantitative sensory testing measures following high-velocity low-amplitude spinal manipulative therapy in musculoskeletal pain populations, in randomised controlled trials. Our objectives were to compare changes in quantitative sensory testing outcomes after spinal manipulative therapy vs. sham, control and active interventions, to estimate the magnitude of change over time, and to determine whether changes are systemic or not.

**Results:**

Fifteen studies were included. Thirteen measured pressure pain threshold, and four of these were sham-controlled. Change in pressure pain threshold after spinal manipulative therapy compared to sham revealed no significant difference. Pressure pain threshold increased significantly over time after spinal manipulative therapy (0.32 kg/cm^2^, CI 0.22–0.42), which occurred systemically. There were too few studies comparing to other interventions or for other types of quantitative sensory testing to make robust conclusions about these.

**Conclusions:**

We found that systemic MIH (for pressure pain threshold) does occur in musculoskeletal pain populations, though there was low quality evidence of no significant difference compared to sham manipulation. Future research should focus on the clinical relevance of MIH, and different types of quantitative sensory tests.

**Trial registration:**

Prospectively registered with PROSPERO (registration CRD42016041963).

## Introduction

### Background

Spinal manipulative therapy (SMT) is commonly utilised by patients seeking relief from spinal pain symptoms [1]. However, the neurophysiologic mechanisms of SMT and the reasons for positive clinical outcomes in some patients is poorly understood. Manipulation-induced hypoalgesia (MIH), a reduction in pain sensitivity following SMT, is one possible explanation. To date, much MIH research has been performed on asymptomatic populations and its clinical relevance is unknown.

Experimental pain research commonly involves quantitative sensory testing (QST). QST comprises a controlled nociceptive stimulus and standardised psycho-physical measurements of the resulting pain [2]. The most widely used QST type in manual therapy research is pressure pain threshold (PPT, which is the detection threshold for deep pain from pressure), though temporal summation (the change in subjective pain intensity during repeated nociceptive stimuli, typically using heat or pinprick), thermal pain detection thresholds, and others, are also used.

It is known that many people with chronic pain have increased pain sensitivity in a variety of QST measures [3, 4]. Clinically, there is poor correlation between pain detection thresholds and subjective pain outcomes (pain intensity and disability), but fair correlations with subjective pain outcomes for pain tolerance thresholds and temporal summation evoked by heat [5].

SMT encompasses a variety of techniques, and sometimes mobilisation is included in the definition. For this review, we are specifically concerned with high-velocity low-amplitude (HVLA) SMT, which involves a rapid, controlled manual thrust targeting specific spinal joints [6]. The thrust is often accompanied by a “cracking” sound (termed cavitation) [6], though hypoalgesia appears to occur regardless of a cavitation [7]. In the following, SMT will refer to HVLA SMT unless specified differently. The mechanism for clinical pain relief associated with SMT is not well understood, though changes in QST measures may offer insight into this. Bialosky et al. [8] argue that the mechanical input of SMT (and manual therapy in general) leads to a neurophysiologic cascade. This may involve peripheral factors (e.g. changes in inflammatory mediators and nociceptors), spinal factors (e.g. altered dorsal horn neuron excitability), and supraspinal factors (e.g. periaqueductal gray activation). Furthermore, it is widely accepted that at least some of the pain relieving effect of SMT is attributable to placebo and contextual factors [8, 9].

### Previous research

Four previous systematic reviews on the topic of MIH conclude that SMT (and mobilisation) leads to increased PPTs (decreased pressure sensitivity) [10–13]. One concludes that this increase in PPT was significant compared to sham in asymptomatic populations [13], and the others do not make conclusions on this topic. A variety of other types of QST measures may also respond to SMT [12], though results for temperature-induced pain are mixed [11, 12]. There is no clear consensus in these reviews regarding whether changes in QST occur only locally, regionally, or systemically in relation to the site of SMT. None of these reviews, however, specifically investigate changes in QST measures after HVLA SMT in musculoskeletal pain populations only.

### Rationale and research questions

Since previous reviews do not adequately address whether MIH occurs in symptomatic populations, and the MIH literature has expanded significantly in recent years, we concluded that an up to date systematic critical review with meta-analysis was warranted. Our overarching aim was to investigate the literature on how HVLA SMT affects short-term QST measures in musculoskeletal pain populations, with the following specific research questions:

1. Is there a difference in change in QST measures after SMT compared to sham or control?
2. Is there a difference in change in QST measures after SMT compared to active interventions?
3. Do QST measures change over time after SMT?
4. Are any changes in QST measures after SMT local, regional, or remote?

## Methods

This review was prospectively registered with PROSPERO, registration number CRD42016041963.

### Eligibility criteria

We included only peer-reviewed randomised controlled trials in English, investigating change in any QST outcome before and after SMT, in human participants with musculoskeletal pain of any type and duration. We considered any year of publication, but arbitrarily limited studies to those with at least 10 participants per group in order to reduce the effect of spurious findings from particularly small studies.

At least one group in each study had to receive HVLA SMT, not combined with any other therapy. SMT could be compared to any other active intervention, sham, or control. Studies had to measure at least one type of QST as a primary or secondary outcome measure, before and after the intervention on the same day.

### Data sources and searches

Studies were identified through a comprehensive literature search of the databases PubMed, Scopus, and CINAHL from inception until December 8, 2016. An additional search was performed on September 21, 2017, to identify any additional articles published in the interim. A manual search of the reference lists of included articles was used to identify any further relevant studies. Since the language used to describe SMT and QST outcome measures is highly variable, we compiled an extensive list of relevant terms. Both lists were used in each search, joined with the Boolean operator ‘AND’. Terms for SMT were:

“spinal manipulative therapy” OR “spinal manipulation” OR “spine manipulation” OR “thrust manipulation” OR “joint manipulation” OR “cervical manipulation” OR “thoracic manipulation” OR “lumbar manipulation” OR “cervicothoracic manipulation” OR “thoracolumbar manipulation” OR “lumbosacral manipulation” OR “sacroiliac manipulation” OR “osteopathic manipulation” OR “chiropractic manipulation” OR “chiropractic adjustment” OR “orthopedic manipulation” OR “musculoskeletal manipulations”

Terms for QST outcomes were:

“pain perception” OR “pain sensitivity” OR “experimental pain” OR “experimental pain sensitivity” OR “experimentally induced pain” OR “experimentally-induced pain” OR “quantitative sensory testing” OR “pain measurement” OR “pain tolerance” OR “pain threshold” OR “pressure pain” OR “pressure pain threshold” OR “pressure sensitivity” OR “pressure pain sensitivity” OR “thermal pain” OR “mechanical pain” OR “exercise-induced pain” OR “electrical pain” OR “chemical pain” OR “pain modulation” OR “analgesia” OR “analgesic” OR “hypoalgesia” OR “hypoalgesic” OR “hyperalgesia” OR “allodynia” OR “algometry” OR “algometer” OR “temporal sensory summation” OR “temporal summation” OR “wind-up” OR “suprathreshold heat response”

### Study selection

Titles and abstracts of search results were screened independently by an author (SA) and an independent reviewer (an academic health professional who was trained in the topic prior to the review) to identify articles for full text retrieval. Full text articles were retrieved based on reviewer agreement, and were screened independently, but unblinded, by two authors (SA and CLY) for inclusion. Disagreements were resolved by consensus between reviewers at each stage, with arbitration by a third author (BW) if required.

### Data extraction and quality assessment

Data were independently extracted by two reviewers from the full text of the included studies using descriptive (SA and CLY), quality (SA and CLY), and results (SA and BW) checklists. The checklists were developed by consensus of two authors (SA and CLY) based on the needs of this review, and were pilot tested on two articles and refined. Any disagreements were resolved by consensus of the two relevant reviewers, and arbitration by a third author if required.

The descriptive information of interest was: 1) study design (number and size of groups, randomised controlled trial or cross-over design), 2) participant information (mean age, age range, sex distribution, type of musculoskeletal pain, source of participants), 3) details on SMT intervention (location, if therapist was allowed to choose target joint, if 2nd thrust was allowed), 4) comparators, and 5) QST outcome measures (type, measured where and when), and 6) area. We used the term ‘area’ to describe the location of the QST measurement in relation to the location of the SMT, considering anatomical and neurological connections. The subgroups used are local, regional, and remote, defined in Table 1. These are based on dermatomal and myotomal patterns, and acknowledging that SMT lacks specificity, affecting multiple joints in the vicinity of the target joint [14, 15]. Convergence of trigeminal nerve and upper cervical afferent inputs in the upper spinal cord has been demonstrated [16], thus we chose to classify the head and face as ‘regional’ in the case of upper cervical SMT.

**Table 1:** Definitions for area of quantitative sensory testing, based on intervention location

Area	Cervical spine SMT	Thoracic spine SMT	Lumbar spine SMT
Local	Cervical spine and paraspinal muscles in close vicinity to SMT location	Thoracic spine and paraspinal muscles in close vicinity to SMT location	Lumbar spine and paraspinal muscles in close vicinity to SMT location
Regional	Upper limb, & head/face if upper/mid cervical SMT	Rest of thorax, e.g. mid/lower trapezius, ribs	Lower limb, pelvis
Remote	Anywhere else	Anywhere else	Anywhere else

Abbreviations: *SMT* Spinal manipulative therapy

Any specific types of QST measured in less than three studies, and studies that measured only these types, were excluded from quality and results tables and from meta-analysis, as conclusions would be difficult to make based on one or two studies. These studies were included in the descriptive table and are briefly discussed in the Results section.

Quality items were based on risk of bias items from the Cochrane Handbook for Systematic Reviews of Interventions [17] and the PRISMA Statement [18]. This approach was deemed appropriate since we were reviewing experimental rather than clinical outcomes. Articles were assigned quality scores out of 12 (maximum one point per item). The quality items, interpretation details, and scoring system are detailed in Table 2.

**Table 2:** Quality items, explanation, and scoring key for included studies

Quality item	Details/explanation	Scoring key (total max. 13)
Was PPT measured correctly, and was reliability pre-tested?	Valid/reliable technique includes taking 3 measures and averaging all 3 or last 2.	Both = 1, Valid technique only = 0.5, Pre-tested reliability only = 0.5, Neither = 0
Was the assessor blinded?	–	Yes = 1, No = 0
Was there appropriate random number generation and concealment?	Random sequence generation, e.g. random number generator. Adequate concealment until randomisation occurs, e.g. sequential opaque envelopes.	Method appropriate = 1, Method for one component but not both reported and appropriate = 0.5, Method NR = 0
Were active and control interventions well described?	–	Yes = 1, No = 0
Were practitioners appropriate and sufficiently experienced?	Practitioner with training in spinal manipulative therapy, ≥3 years clinical experience.	Yes = 1, No/NR = 0
Were attempts made to keep participants naïve to study aims? If sham-controlled, were they blinded, and confirmed?	*If sham group:* Blinded? Confirmed? Naïve to study aims? *If no sham group:* Naïve to study aims?	*If sham:* Blinding confirmed = 1, Blinding attempted but not confirmed and/or naïve = 0.5, Not blind/naïve = 0 *If no sham:* Naïve = 1, Not naïve/NR = 0
Were study conditions controlled?	An effort to control temperature, room, interactions, expectations	Yes = 1, No/NR = 0
Was there control for psychosocial modifiers/confounders?	Statistical control	Yes = 1, No = 0
Was a sample size calculation performed and met?	Performed based on PPT estimates?	Yes = 1, Performed and met but NR based on what = 0.5, Not performed, not performed based on PPT, or not met = 0
Were losses and exclusions reported clearly?	–	Yes = 1, No = 0
Missing data reported? Imputation method reported and appropriate, if required?	–	Yes = 1, No/NR = 0
Were estimates and *p*-values/CIs reported for between-group differences?	–	Yes = 1, Estimates/CIs NR but *p*-values non-significant = 1, No = 0

Abbreviations: *CIs* Confidence intervals, *NR* Not reported, *PPT* Pressure pain threshold

Two working results tables were constructed, for within-group change and between-group differences respectively (not presented). Within-group results are reported for SMT groups only. PPT is the only QST measure reported in the results tables, and is reported as actual and percentage change from baseline. Percentage change is helpful for interpretation, since absolute values can vary widely based on testing location [19]. Between-group results are reported as the difference in the mean change between SMT and comparator groups. Data were converted to kg/cm^2^ where relevant, and we calculated change in PPT and between-group differences based on data presented in the full text of the study (when required and if possible). Included in these tables were relevant descriptive information, statistical significance of results, and the articles’ quality score.

### Data synthesis and interpretation

The working results tables were colour coded based on the statistical significance of the results (alpha level .05). These working tables were used systematically to answer the research questions. These data are presented as a single results table without colour coding in this article, Table 5. Studies are ordered in the results table based on quality (highest to lowest). Since items related to risk of bias are included in the quality table, risk of bias was not considered separately. During the interpretation, we assessed whether studies of lower quality generally agreed or disagreed with studies of higher quality. This assisted in determining the weight to place on a result.

#### Meta-analyses

Meta-analyses were performed with Comprehensive Meta-Analysis V3 (Biostat, Inc., USA) software, using mean change from baseline and standard deviation (SD) of the change in each group. If an intervention group in a single study had two or more testing sites eligible for inclusion in a given meta-analysis, a combined mean change and variance was calculated, as recommended in Borenstein et al. [20]. This was in order to account for lack of independence with multiple outcome measures. The calculation for combined variance requires assuming a correlation between the outcomes being combined. Since we could not identify any published estimates of the correlation between PPT at different testing sites, we chose to run a sensitivity analysis by calculating the variances twice, based on high and low assumed correlations (0.75 and 0.25 respectively), and compare meta-analysis results. Studies were not excluded based on quality scores. Given the heterogeneity in testing sites, specific interventions, and study populations, analyses were run under a random effects model. Heterogeneity was assessed with I^2^, with > 75% indicating considerable heterogeneity between studies [17].

If at least three studies included in the meta-analyses utilised repeated measures post-intervention with at least 15 min follow-up, we planned to analyse the data by groups of time points as appropriate, based on the spread of these time points. Otherwise we intended to use the first post-intervention measurement for all studies.

We planned to perform the following meta-analyses:

1. Mean change from baseline of all SMT groups and testing area
  - Subgroup analyses: mean change from baseline for each testing area (local, regional, and remote)
2. Difference between SMT and each type of comparator (minimum three studies)

Numerous studies did not provide SDs of the change, but provided other data that allowed calculation of SDs as follows. If mean change from baseline with either 95% confidence intervals (CIs) or standard error were provided, SDs of the change were calculated based on formulae from *section 7.7.3.2 Obtaining standard deviations from standard errors and confidence intervals for group means* of the Cochrane Handbook [17].

Several studies separated results into right and left or ipsilateral and contralateral results at each testing site. Sides were combined by averaging the mean of each side, and using a formula to calculate combined SDs of the change, provided in *table7.7.a Formulae for combining groups* in the Cochrane Handbook [17]. One study [21] reported data separately for participants who received right and left cervical SMT. These groups were also combined using the same formula, since we were not interested in side to side differences of MIH and the study reported there were no differences between groups.

## Results

**Fig. 1:**
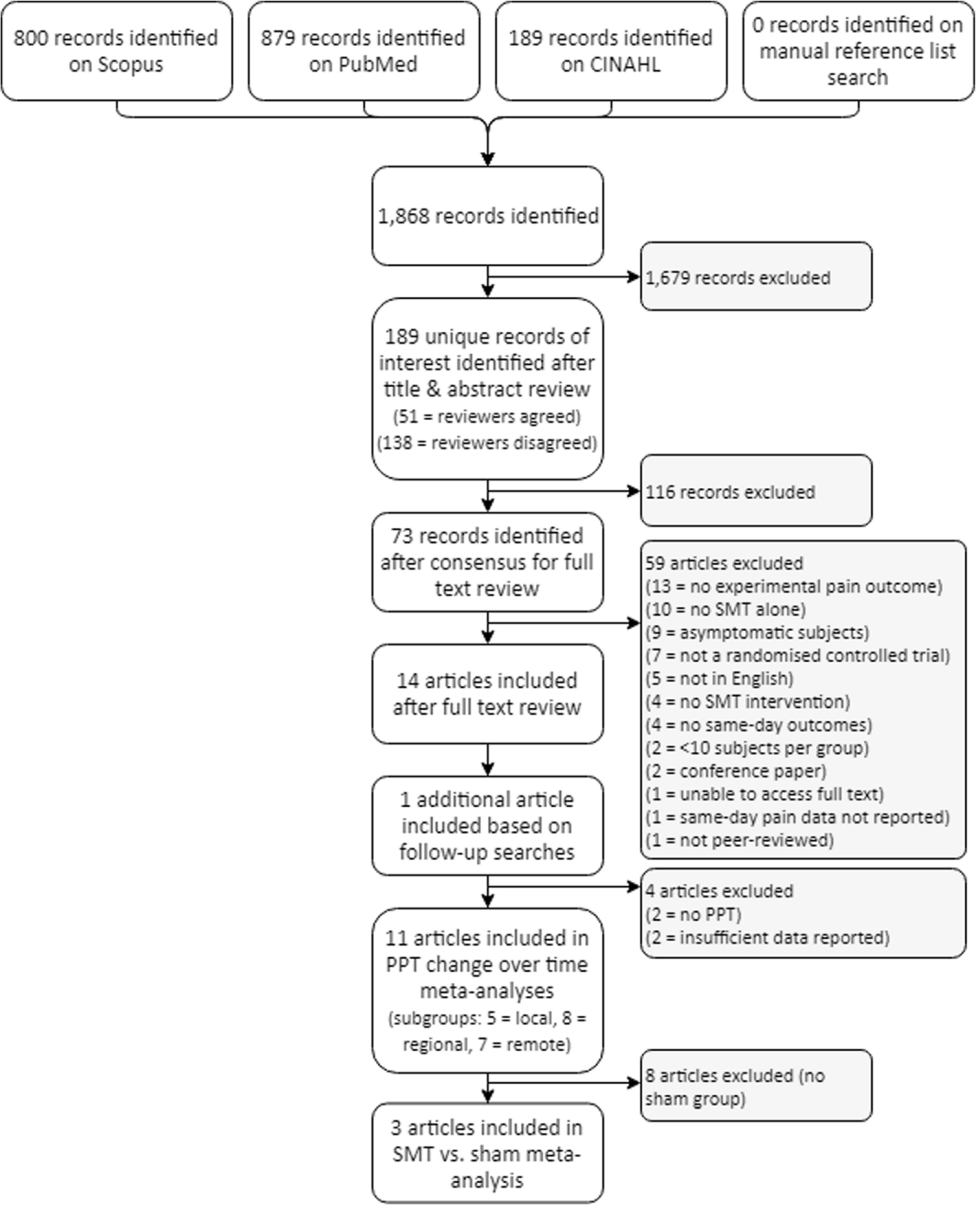
Study selection flow chart. Abbreviations: SMT = spinal manipulative therapy

A total of 1868 records were identified in the initial search, completed on December 8, 2016, with none added after reference list searches. Seventy-three articles were retrieved for full text review, with 14 identified as meeting inclusion criteria. The follow-up search on September 21, 2017, identified one additional article. PPT was the only type of QST measured in three or more studies, thus only PPT was addressed in quality and results tables and meta-analyses. The tables were adapted to specifically suit PPT, excluding two studies did not measure PPT [22, 23]. Two further studies were excluded from meta-analyses due to insufficient data reporting [24, 25]. See Fig. 1.

### Study characteristics

Table 3 contains a full description of the 15 included studies’ characteristics.

**Table 3:** Descriptions of studies included in systematic review

1st author, year, country	Design	No. of participants	Age (range)	Male/ Female	Musculoskeletal pain type	Source of participants	SMT	Control or comparison/s	Outcome measure/s	PPT area	Time of follow-up
Bautista-Aguirre 2017, Spain [27]	3 arm RCT	88 (28/30/30)	32 (NR)	24/64	Chronic non-specific NP	Primary care	1. C7 HVLA SMT 2. T3 HVLA SMT (NR if 2nd allowed)	Control (manual contact to head for 3 min)	PPT @ median nerve at wrist & elbow, ulnar nerve at elbow, radial nerve at upper arm	1. Regional 2. Remote	Immediate
Bialosky 2009, USA [22]	3 arm RCT	36 (12/12/12)	32 (NR)	10/26	Non-specific LBP	General population & primary care	Bilateral SIJ HVLA SMT (2 regardless of cavitation)	1. Stationary bicycle (5 min) 2. Lumbar extension exercises	TS @ palmar hand & plantar foot Aδ “first” pain @ volar forearm & calf	*NA*	Immediate
Bialosky 2014, USA [23]	4 arm RCT	110 (28/27/27/28)	32 (NR)	33/77	Non-specific LBP	General population & primary care (not direct referral)	Bilateral SIJ HVLA SMT (2 regardless of cavitation)	1. Sham SMT 2. Sham SMT + positive expectation 3. Control (5 min quiet sitting)	Mechanical pain sensitivity @ PSIS & 1st web-space of foot Suprathreshold heat response @ plantar foot Aftersensations @ plantar foot	*NA*	Immediate
Casanova-Méndez 2014, Spain [28]	2 arm RCT	60 (30/30)	38 (NR)	17/43	Chronic non-specific NP	Primary care	1. T4 supine HVLA SMT 2. T4 toggle-recoil HVLA SMT (2nd if no cavitation)	*Nil*	PPT @ C4 SP, T4 SP, & upper trapezius	Local & remote	Immediate 20 min
Coronado 2015, USA [33]	3 arm RCT	78 (26/27/25)	39 (NR)	42/36	General shoulder pain	General population & primary care	Mid-cervical HVLA SMT (NR if pre-specified, 2nd if no cavitation)	1. Shoulder manipulation 2. Shoulder exercise program	PPT @ acromion & tibialis anterior HPT @ volar forearm TS @ volar forearm	Regional & remote	Immediate 1–2 wks
Côté 1994, Canada [25]	2 arm RCT	30 (16/14)	31 (22–50)	16/14	Chronic non-specific LBP	Primary care	L5-S1 or SIJ HVLA SMT (therapist-chosen level, NR if 2nd allowed)	Lumbar mobilisation	PPT @ L5 paraspinals, SIJ, & upper gluteal muscles	Local & regional	Immediate 15 min 30 min
De Oliveira 2013, Brazil [32]	2 arm RCT	148 (74/74)	46 (NR)	39/109	Chronic non-specific LBP	General population & primary care	1. “Global” T1-T5 HVLA SMT (pre-specified) 2. Lumbar HVLA SMT (therapist-chosen level) (NR if 2nd allowed)	*Nil*	PPT @ L3 & L5 paraspinals, & tibialis anterior	1. Remote 2. Local & regional	Immediate
Fernández-Carnero 2008, Spain [34]	2 arm cross-over RCT	10	42 (30–49)	5/5	Lateral epicondylalgia	General population	C5–6 HVLA SMT (2nd if no cavitation)	Sham SMT	PPT @ lateral epicondyle HPT @ lateral epicondyle CPT @ lateral epicondyle	Regional	5 min
Kardouni 2015, USA [35]	2 arm RCT	45 (24/21)	31 (18–59)	22/23	Shoulder pain	General population & primary care	Thoracic HVLA SMT (NR if pre-specified, 2 thrusts in each of lower, mid, and upper)	Sham SMT	PPT @ deltoid & lower trapezius	Regional & remote	Immediate
Lopez-Lopez 2015, Spain [29]	3 arm RCT	48 (15/16/17)	35/36/37 by group (NR)	6/42	Chronic non-specific NP	Primary care	Cervical HVLA SMT (therapist-chosen level, 2nd if no cavitation)	1. P-A grade III mobilisation 2. Sustained apophyseal natural glide mobilisation	PPT @ C2 SP	Local	5 min
Maduro de Camargo 2011, Brazil [30]	2 arm RCT	37 (17/20)	31/28 by group (19–45)	21/16	Non-specific NP	General population	C5–6 HVLA SMT (2nd if no cavitation)	Control (2 min quiet sitting)	PPT @ upper trapezius, deltoid, & C5 SP	Local & regional	5 min
Mansilla-Ferragut 2009, Spain [26]	2 arm RCT	37 (18/19)	35 (21–50)	0/37	Chronic non-specific NP	General population	Bilateral C0–1 HVLA SMT (2nd if no cavitation)	Sham SMT	PPT @ temple	Regional	5 min
Martínez-Segura 2012, Spain [21]	3 arm RCT	90 (29/28/33)	37 (NR)	44/46	Chronic non-specific NP	Primary care	1. Right mid-cervical HVLA SMT 2. Left mid-cervical HVLA SMT 3. Broad T1-T4 HVLA SMT (therapist-chosen level, 2nd if no cavitation)	*Nil*	PPT @ C5–6 facet, lateral epicondyle, & tibialis anterior	1 & 2. Local, regional & remote 3. Remote	10 min
Packer 2014, Brazil [24]	2 arm RCT	32 (16/16)	23/26 by group (21–29)	0/32	Chronic temporo-mandibular disorder & non-specific NP	NR	T1 HVLA SMT (2nd if no cavitation)	Sham SMT	PPT @ masseter, temporalis, & temporomandibular joint	Remote	Immediate 48–72 h
Salom-Moreno 2014, Spain [31]	2 arm RCT	52 (27/25)	33 (NR)	22/30	Chronic non-specific NP	Primary care	T3-T6 HVLA SMT (2nd if no cavitation)	T3-T6 grade III-IV mobilisation	PPT @ C5–6 facet, 2nd metacarpal, & tibialis anterior	Remote	10 min

Abbreviations: *CPT* Cold pain threshold, *HPT* Heat pain threshold, *HVLA* High-velocity low-amplitude, *LBP* Low back pain, *NA* Not applicable, *NP* Neck pain, *NR* Not reported, *P-A* Posterior to anterior, *PPT* Pressure pain threshold, *PSIS* Posterior sacroiliac spine, *RCT* Randomised controlled trial, *SIJ* Sacroiliac joint, *SMT* Spinal manipulative therapy, *SP* Spinous process, *TS* Temporal summation

#### Populations

The weighted mean age of participants was 35.9 years (mean age range 23–46 years), with a total of 901 participants across all studies. Of these, 600 (66.7%) were female. Two studies included only female participants [24, 26].

There was a mixture of spinal pain and pain in other areas; seven non-specific neck pain [21, 26–31], four non-specific low back pain [22, 23, 25, 32], one temporomandibular disorder with non-specific neck pain [24], and three extremity pain [33–35]. Nine studies where performed on chronic pain populations [21, 24–29, 31, 32], and six on study samples with mixed or unknown chronicity [22, 23, 30, 33–35].

#### Quantitative sensory testing outcomes

Thirteen studies utilised PPT as an outcome measure [21, 24–35]. Temporal summation was measured in two studies [22, 33], but pre-post intervention scores were not analysed in one of these [33]. Heat pain detection threshold was measured in two studies [33, 34]. Aδ “first” pain (subjective rating of “first” pain in response to an increasing heat stimulus) [22], suprathreshold mechanical pain sensitivity (subjective pain rating of a standard pressure stimulus) [23], suprathreshold heat response (subjective rating of “second” pain for the final stimulus of a series of five heat stimuli) [23], aftersensations (subjective rating of pain 15 s after a series of heat stimuli) [23], and cold pain detection threshold [34] were each measured in single studies.

#### Interventions

While each study included at least one SMT group, there was a range of comparators in PPT studies. Four studies compared SMT to sham [24, 26, 34, 35], two compared to a passive control [27, 30], three compared to mobilisation [25, 29, 31], one compared to exercise [33], one to extremity manipulation [33], and four to another SMT [21, 27, 28, 32]. The studies without PPT compared SMT to exercise [22], sham [23], and control conditions [23].

#### Follow-up

Seven studies measured PPT immediately after intervention [24, 25, 27, 28, 32, 33, 35], with two of these also taking same-day measures at least 15 min post-intervention [25, 28]. Four studies measured five minutes post-intervention [26, 30, 34, 35], and two studies measured 10 min post-intervention [21, 31]. Two studies also measured outcomes on a different day [24, 33], but those data are not considered in this review. The studies without PPT both measured immediately post-intervention [22, 23].

#### Other factors

How studies determined the target vertebral joints for SMT was variable. Nine studies pre-defined the target joint [22–24, 26–28, 30, 31, 34], while three allowed the treating practitioner to choose a target joint (within a region) based on history and examination findings [21, 25, 29]. One study used each approach for each of two SMT groups [32], and two studies did not report adequately on this topic [33, 35]. Nine studies allowed practitioners to deliver a second SMT thrust if a cavitation was not achieved on the first thrust [21, 24, 26, 28–31, 33, 34]. Three studies delivered a fixed number of thrusts [22, 23, 35], and three did not report on this [25, 27, 32].

In those measuring PPT, six studies measured locally [21, 25, 28–30, 32], nine regionally [21, 25–27, 30, 32–35], and eight remotely [21, 24, 27, 28, 31–33, 35]. There was considerable variation in testing sites between studies.

### Quality of studies

See Table 4 for quality items and scores for the 13 studies measuring PPT. Articles were scored out of 12. Mean and median quality scores were 6.6 (SD 1.2) and 6.5 respectively, with a range of 4.5–8. We chose to group articles post-hoc as lower, moderate, and higher quality, arbitrarily using scores of 4.5–5.5, 6–7, and 7.5–8 respectively as cut-points to assist discussion.

**Table 4:** Quality items and quality scoring of studies using pressure pain threshold

1st author, year	Was PPT measured correctly, & was reliability pre-tested?	Was the assessor blinded?	Was there appropriate random number generation & concealment?	Were active & control interventions well described?	Were practitioners appropriate & sufficiently experienced?	Were attempts made to keep participants naïve to study aims? If sham-controlled, were they blinded, & confirmed?	Were study conditions controlled?	Was there control for psychosocial modifiers/ confounders?	Was a sample size calculation performed & met?	Were losses and exclusions reported clearly?	Missing data reported? Imputation method reported & appropriate, if required?	Were estimates & *p*-values/CIs reported for between-group differences?	Quality score
Casanova-Méndez 2014 [28]	Technique ✔, not pre-tested (0.5)	Yes (1)	Yes (1)	Yes (1)	Yes (1)	Were naïve (1)	NR (0)	No (0)	Calculation done & met, NR based on what (0.5)	Yes (1)	NR (0)	Yes (1)	8
Coronado 2015 [33]	Technique ✔, not pre-tested (0.5)	Yes (1)	Yes (1)	Yes (1)	Yes (1)	Not naïve (0)	NR (0)	No (0)	Calculation done & met, NR based on what (0.5)	Yes (1)	Yes, acceptably imputed (1)	Yes (1)	8
Kardouni 2015 [35]	Technique ✔, pre-testing found reliable (1)	Yes (1)	Yes (1)	Yes (1)	Yes (1)	Blinding confirmed effective (1)	NR (0)	No (0)	Calculation done, NOT met (0)	Yes (1)	NR (0)	Yes (1)	8
Bautista-Aguirre 2017 [27]	Technique ✔, not pre-tested (0.5)	Yes (1)	Yes (1)	Yes (1)	Yes (1)	Not naïve (0)	NR (0)	No (0)	Calculation done & met (1)	Yes (1)	NR (0)	Yes (1)	7.5
Martínez-Segura 2012 [21]	Technique ✔, not pre-tested (0.5)	Yes (1)	Yes (1)	Yes (1)	Yes (1)	Not naïve (0)	NR (0)	No (0)	Calculation done & met (1)	Yes (1)	NR (0)	Yes (1)	7.5
De Oliveira 2013 [32]	Technique ✔, pre-testing found reliable (1)	Yes (1)	Yes (1)	Yes (1)	Yes (1)	Not naïve (0)	NR (0)	No (0)	Calculation based on subjective pain intensity (0)	Yes (1)	NR (0)	Yes (1)	7
Maduro de Camargo 2011 [30]	Technique ✔, not pre-tested (0.5)	Yes (1)	Method NR (0)	Yes (1)	Yes (1)	Were naïve (1)	NR (0)	No (0)	Calculation done & met (1)	No (0)	NR (0)	Yes (1)	6.5
Packer 2014 [24]	NR if PPT tested × 3, not pre-tested (0)	Yes (1)	Yes (1)	Yes (1)	NR (0)	Blinded, not checked, NR if naïve (0.5)	NR (0)	No (0)	Calculation done & met (1)	Yes (1)	NR (0)	Yes (1)	6.5
Salom-Moreno 2014 [31]	Technique ✔, not pre-tested (0.5)	Yes (1)	Yes (1)	Yes (1)	NR (0)	Not naïve (0)	NR (0)	No (0)	Calculation done & met (1)	Yes (1)	NR (0)	Yes (1)	6.5
Côté 1994 [25]	NR if PPT tested × 3, not pre-tested (0)	Yes (1)	Concealment method NR (0.5)	Yes (1)	NR (0)	Not naïve (0)	NR (0)	No (0)	Calculation done & met (1)	Yes (1)	NR (0)	*p*-value non-significant, estimates & CIs NR (1)	5.5
Lopez-Lopez 2015 [29]	Technique ✔, not pre-tested (0.5)	Yes (1)	Yes (1)	Yes (1)	NR (0)	Not naïve (0)	NR (0)	Yes (1)	Calculation based on subjective pain intensity (0)	Yes (1)	NR (0)	No (0)	5.5
Mansilla-Ferragut 2009 [26]	Technique ✔, not pre-tested (0.5)	Yes (1)	Concealment method NR (0.5)	Yes (1)	Yes (1)	Not blinded, not naïve (0)	NR (0)	No (0)	No calculation (0)	No (0)	NR (0)	Yes (1)	5
Fernández-Carnero 2008 [34]	Technique ✔, not pre-tested (0.5)	Yes (1)	Method NR (0)	Yes (1)	Yes (1)	Not blinded, not naïve (0)	NR (0)	No (0)	No calculation (0)	No (0)	NR (0)	Yes (1)	4.5

Note: points given per item in brackets. Abbreviations: ✔ Correct, *CIs* Confidence intervals, *NR* Not reported, *PPT* Pressure pain threshold

### Answers to research questions

#### Is there a difference in PPT comparing SMT to sham, or SMT to control?

See Table 5 for results from the 13 studies measuring PPT. Out of four sham-controlled studies, two found no significant differences in change in PPT between SMT and sham groups. One of these was higher quality with confirmed blinding of participants [35], and the other was moderate quality with attempted but unconfirmed blinding [24]. The remaining two studies found a significant increase in PPT after cervical SMT compared to sham [26, 34]. These were both of lower quality, with no reported attempt to blind participants.

**Table 5:** Results for within-group change and between-group differences in pressure pain threshold

1st author, year	Groups	PPT testing site/s & follow-up time/s	Within-group estimates; mean change from baseline in kg/cm^2^ (SD, % change)	Significant within-group change (SMT groups only)?^	Difference in mean change in kg/cm^2^ [group 1 minus group 2]	Significant between-group difference?^	Quality score
Casanova-Méndez, 2014 [28]	Supine TSMT (TSMT 1), Toggle-recoil TSMT (TSMT 2)	C4 SP, T4 SP, & upper trapezius,^a^ immediately & 20 mins after intervention	*C4 SP*		*TSMT 1 vs TSMT 2*		8
			SMT 1 immed.: 0.21 (0.48, 10.4%)	Yes	C4 SP immed.: 0.00	No	
			SMT 1 20 min: 0.25 (0.59, 12.4%)	Yes	C4 SP 20 min: − 0.05	No	
			SMT 2 immed.: 0.21 (0.32, 10.7%)	Yes	T4 SP immed.: 0.03	No	
			SMT 2 20 min: 0.30 (0.42, 15.3%)	Yes	T4 SP 20 min: − 0.23	No	
			*T4 SP*		Trapezius immed.: − 0.19	No	
			TSMT 1 immed.: 0.40 (0.59, 10.8%)	Yes	Trapezius 20 min: − 0.11	No	
			TSMT 1 20 min: 0.47 (0.76, 12.7%)	Yes			
			TSMT 2 immed.: 0.37 (0.67, 11.0%)	Yes			
			TSMT 2 20 min: 0.70 (0.79, 20.9%)	Yes			
			*Trapezius*				
			TSMT 1 immed.: 0.22 (0.60, 6.7%)	Yes^c^			
			TSMT 1 20 min: 0.32 (0.80, 9.7%)	Yes^c^			
			TSMT 2 immed.: 0.41 (0.51, 13.6%)	Yes^c^			
			TSMT 2 20 min: 0.43 (0.53, 14.2%)	Yes^c^			
Coronado, 2015 [33]	CSMT, Shoulder manipulation, Shoulder exercises	Acromion & tibialis anterior, immediately after intervention	*Acromion*		*CSMT vs Shoulder manipulation*		8
			CSMT: 0.37 (0.68, 14.2%)	Yes^c^	Acromion: 0.18	No	
			Shoulder manipulation: 0.19 (0.70, 5.6%)	-	Tibialis anterior: 0.26	No	
			Exercise: 0.10 (0.69, 3.1%)	-	*CSMT vs Exercise*		
			*Tibialis anterior*		Acromion: 0.27	No	
			CSMT: 0.35 (0.84, 7.4%)	Yes^c^	Tibialis anterior: 0.10	No	
			Shoulder manipulation: 0.09 (0.83, 1.7%)	-			
			Exercise: 0.25 (1.89, 4.5%)	-			
Kardouni, 2015 [35]	TSMT, Sham SMT	Deltoid^a^ & lower trapezius,^a^ immediately after intervention	*Deltoid*		*TSMT vs Sham*		8
			TSMT: 0.10 (1.65, 2.8%)	No	Deltoid: 0.11	No	
			Sham: − 0.01 (1.51, − 0.1%)	-	Trapezius: 0.11	No	
			*Trapezius*				
			TSMT: 0.09 (1.70, 2.2%)	No			
			Sham: − 0.02 (1.49, − 0.3%)	-			
Bautista-Aguirre, 2017 [27]	CSMT, TSMT, Control	Median nerve (wrist),^a^ median nerve (elbow),^a^ ulnar nerve,^a^ & radial nerve,^a^ immediately after intervention	*Median nerve (wrist)*		*CSMT vs control*		7.5
			CSMT: 0.24 (1.41, 3.4%)	No^c^	Median nerve (wrist): 0.29	No	
			TSMT: 0.27 (1.38, 3.9%)	No^c^	Median nerve (elbow): 0.14	No	
			Control: − 0.05 (3.52, − 0.8%)	-	Ulnar nerve: 0.26	No	
			*Median nerve (elbow)*		Radial nerve: 0.44	No	
			CSMT: 0.2 (0.72, 6.8%)	No^c^	*TSMT vs control*		
			TSMT: 0.2 (0.7, 6.8%)	No^c^	Median nerve (wrist): 0.33	No	
			Control: 0.07 (0.91, 2.6%)	-	Median nerve (elbow): 0.14	No	
			*Ulnar nerve*		Ulnar nerve: 0.25	No	
			CSMT: 0.38 (1.01, 9.2%)	No^c^	Radial nerve: 0.42	No	
			TSMT: 0.37 (0.99, 9.1%)	No^c^	*CSMT vs TSMT*		
			Control: 0.11 (0.7, 2.8%)	-	Median nerve (wrist): − 0.03	No	
			*Radial nerve*		Median nerve (elbow): 0.01	No	
			CSMT: 0.83 (1.25, 24.1%)	Yes^c^	Ulnar nerve: 0.01	No	
			TSMT: 0.81 (1.21, 23.6%)	Yes^c^	Radial nerve: 0.02	No	
			Control: 0.39 (0.82, 11.3%)	-			
Martínez-Segura, 2012 [21]	CSMT,^b^ TSMT	C5–6 facet,^a^ lateral epicondyle,^a^ & tibialis anterior,^a^ 10 mins after intervention	*C5–6 facet*		*CSMT vs TSMT*		7.5
			CSMT: 0.40 (0.33, 29.1%)	Yes	C5–6 facet: 0.10	No	
			TSMT: 0.30 (0.32, 23.1%)	Yes	Lateral epicondyle: 0.05	No	
			*Lateral epicondyle*		Tibialis anterior: 0.07	No	
			CSMT: 0.45 (0.46, 31.6%)	Yes			
			TSMT: 0.40 (0.36, 28.6%)	Yes			
			*Tibialis anterior*				
			CSMT: 0.82 (0.67, 38.8%)	Yes			
			TSMT: 0.75 (0.46, 36.6%)	Yes			
De Oliveira, 2013 [32]	“Non-region specific” TSMT, “Region-specific” LSMT	Lumbar paraspinals & tibialis anterior, immediately after intervention	*Paraspinals*		*TSMT vs LSMT*		7
			TSMT: 0.37 (1.36, 7.5%)	Yes	Paraspinals: 0.18	No	
			LSMT: 0.19 (1.53, 3.8%)	No	Tibialis anterior: − 0.12	No	
			*Tibialis anterior*				
			TSMT: 0.11 (1.26, 1.7%)	No			
			LSMT: 0.23 (1.12, 3.6%)	No			
Maduro de Camargo, 2011 [30]	CSMT, Control	Upper trapezius,^a^ deltoid,^a^ & C5 SP, 5 mins after intervention	*Trapezius*		*CSMT vs Control*		6.5
			CSMT: 0.3 (0.41, 9.0%)	Yes^c^	Trapezius: 0.10	No	
			Control: 0.2 (0.69, 5.7%)	-	Deltoid: 0.45	Yes	
			*Deltoid*		C5 SP: 0.20	Yes	
			CSMT: 0.25 (0.49, 7.7%)	Yes^c^			
			Control: − 0.2 (0.58, − 6.3%)	-			
			*C5 SP*				
			CSMT: 0.1 (0.19, 4.3%)	Yes^c^			
			Control: − 0.1 (0.53, − 4.3%)	-			
Packer, 2014 [24]	TSMT, Sham SMT	TMJ,^a^ masseter,^a^ & temporalis,^a^ immediately after intervention	*TMJ*		*TSMT vs Sham*		6.5
			TSMT: − 0.05 (NR, − 7.7%)	No	TMJ: − 0.05	No	
			Sham: 0.00 (NR, 0.0%)	-	Masseter: 0.05	No	
			*Masseter*		Temporalis: 0.00	No	
			TSMT: 0.05 (NR, 10.0%)	No			
			Sham: 0.00 (NR, 0.0%)	-			
			*Temporalis*				
			TSMT: 0.00 (NR, 0.0%)	No			
			Sham: 0.00 (NR, 0.0%)	-			
Salom-Moreno, 2014 [31]	TSMT, Thoracic mob.	C5–6 facet,^a^ 2^nd^ metacarpal,^a^ & tibialis anterior,^a^ 10 mins after intervention	*C5–6 facet*		*TSMT vs Mob.*		6.5
			TSMT: 0.37 (0.17, 27.2%)	Yes	C5–6 facet: 0.26	No	
			Mob.: 0.11 (0.20, 7.4%)	-	2^nd^ metacarpal: 0.03	No	
			*2* ^*nd*^ *metacarpal*		Tibialis anterior: 0.00	No	
			TSMT: 0.20 (0.12, 7.4%)	Yes			
			Mob.: 0.16 (0.43, 6.4%)	-			
			*Tibialis anterior*				
			TSMT: 0.09 (0.45, 2.1%)	Yes			
			Mob.: 0.09 (0.10, 2.2%)	-			
Côté, 1994 [25]	LSMT, Lumbar mob.	L5 paraspinals, SIJs, & gluteal muscles, immediately, 15 mins & 30 mins after intervention	*L5 paraspinals*		*LSMT vs Mob.*		5.5
			LSMT immed.: 0.00 (NR, 0.0%)	No	L5 paraspinals immed.: 0.65	No	
			LSMT 15 min: − 0.11 (NR, − 2.1%)	No	L5 paraspinals 15 min: 0.65	No	
			LSMT 30 min: 0.20 (NR, 3.7%)	No	L5 paraspinals 30 min:1.07	No	
			Mob. immed.: − 0.65 (NR, − 11.0%)	-	SIJs immed.: 0.95	No	
			Mob. 15 min: − 0.77 (NR, − 12.9%)	-	SIJs 15 min: 0.45	No	
			Mob. 30 min: − 0.87 (NR, − 14.7%)	-	SIJs 30 min: 1.10	No	
			*SIJs*		Gluteals immed.: 0.32	No	
			LSMT immed.: 0.66 (NR, 12.9%)	No	Gluteals 15 min: 0.12	No	
			LSMT 15 min: 0.09 (NR, 1.7%)	No	Gluteals 30 min: 0.44	No	
			LSMT 30 min: 0.53 (NR, 10.4%)	No			
			Mob. immed.: − 0.29 (NR, − 4.7%)	-			
			Mob. 15 min: − 0.36 (NR, − 6.0%)	-			
			Mob. 30 min: − 0.57 (NR, − 9.4%)	-			
			*Gluteals*				
			LSMT immed.: 0.31 (NR, 6.1%)	No			
			LSMT 15 min: 0.44 (NR, 8.9%)	No			
			LSMT 30 min: 0.59 (NR, 11.9%)	No			
			Mob. immed.: − 0.02 (NR, − 0.3%)	-			
			Mob. 15 min: 0.32 (NR, 6.4%)	-			
			Mob. 30 min: 0.16 (NR, 3.1%)	-			
Lopez-Lopez, 2015 [29]	CSMT, P-A cervical mob. (mob. 1), glide cervical mob. (mob. 2)	C2 SP, 5 mins after intervention	*C2 SP*		*CSMT vs Mob. 1*		5.5
			CSMT: 0.08 (0.43, 4.6%)	Yes	C2 SP: −0.13	No	
			Mob. 1: 0.21 (0.40, 14.1%)	-	*CSMT vs Mob. 2*		
			Mob. 2: 0.30 (0.41, 18.0%)	-	C2 SP: − 0.22	No	
Mansilla Ferragut, 2009 [26]	CSMT, Sham SMT	Sphenoid bone, 5 mins after intervention	*Sphenoid bone*		*CSMT vs Sham*		5
			CSMT: 0.1 (0.20, 12.5%)	Yes^c^	Sphenoid bone: 0.20	Yes	
			Sham: − 0.1 (0.30, − 12.5%)	-			
Fernández-Carnero, 2008 [34]	CSMT, Sham SMT	Lateral epicondyle,^a^ 5 mins after intervention	*Lateral epicondyle*		*CSMT vs Sham*		4.5
			CSMT: 1.00 (0.68, 27.9%)	Yes	Lateral epicondyle: 0.90	Yes	
			Sham: 0.10 (0.45, 2.5%)	-			

Abbreviations: *CSMT* Cervical SMT, *HVLA* High-velocity low-amplitude, *immed.* Immediate, *LSMT* Lumbosacral SMT, *mob.* Mobilisation, *NR* Not reported (and unable to calculate), *PPT* Pressure pain threshold, *SD* Standard deviation, *SIJ* Sacroiliac joint, *SP* Spinous process, *SMT* Spinal manipulative therapy, *TSMT* Thoracic SMT, *TMJ* Temporomandibular joint

^ *p* < .05

^a^Right and left side data combined by reviewers

^b^Right and left cervical SMT groups’ data combined by reviewers

^c^Significance inferred from 95% confidence interval that does not cross zero

**Fig. 2:**
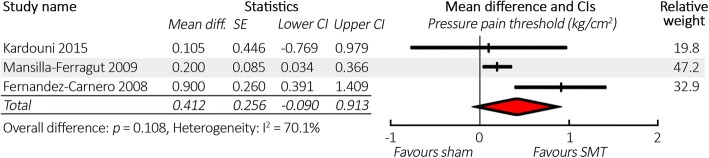
Forest plot comparing spinal manipulative therapy to sham for change in pressure pain threshold, in descending order of study quality. Abbreviations: CI = 95% confidence interval, SE = standard error, SMT = spinal manipulative therapy, CSMT = cervical SMT, TSMT = thoracic SMT, LSMT = lumbar SMT. Note: using correlation of 0.75 for calculations of combined variance

Three studies were included in the meta-analysis comparing change in PPT between SMT and sham (*N* = 92). There was minimal difference in results of the meta-analysis whether we assumed a correlation of 0.75 or 0.25 for calculations of combined variance. We will discuss results based on the more correlation of 0.75, which gives slightly more conservative results. There was no significant difference in the mean change in PPT after SMT compared to sham (0.41 kg/cm^2^, CI -0.09 – 0.91). See Fig. 2 for a forest plot. Full results for all meta-analyses (including sensitivity analysis) are reported in Table 6.

**Table 6:** Full results of senstivity analyses

Meta-analysis	Correlation for variance calculations^a^	Mean kg/cm^2^	Hedge’s *g*	Standard error	95% confidence interval	*p*-value	I^2^	Sample size
Change over time after SMT (all areas)	0.75 0.25	0.320 0.320	0.238 0.248	0.051 0.049	0.220–0.421 0.224–0.416	.000 .000	82.5% 85.5%	693
Local subgroup	0.75 0.25	0.259 0.259	0.169 0.169	0.078 0.078	0.106–0.412 0.106–0.412	.001 .001	81.7% 81.7%	383
Regional subgroup	0.75 0.25	0.349 0.348	0.178 0.184	0.085 0.082	0.181–0.516 0.187–0.509	.000 .000	79.8% 80.1%	533
Remote subgroup	0.75 0.25	0.374 0.372	0.216 0.231	0.073 0.068	0.230–0.517 0.238–0.506	.000 .000	84.1% 86.0%	561
SMT vs. Sham difference	0.75 0.25	0.412 0.398	0.166 0.169	0.256 0.243	−0.090 - 0.913 −0.079 - 0.875	.108 .102	70.1% 70.3%	92

^a^This is the assumed correlation used in calculations for combined variances in studies with multiple testing sites. See Methods for explanation

Two studies compared SMT against control. The higher quality study found no significant difference in change in PPT between two SMT groups and a control condition with manual contact to the head [27]. The moderate quality study found a significant increase in PPT after cervical SMT compared to a control of quiet sitting, at two of three testing sites [30].

#### Is there a difference in PPT comparing SMT to mobilisation or other therapy?

Three studies compared changes in PPT between SMT and mobilisation. Each found no significant differences between groups. One study was moderate [31] and two were lower [25, 29] quality. One of the three studies provided insufficient data for use in meta-analysis [25], thus a meta-analysis was not performed.

Four studies compared changes in PPT in two different SMT groups, with no significant differences between SMT comparisons. One study compared two different HVLA techniques in the same spinal region [28], and three studies compared SMT in different regions of the spine [21, 27, 32]. Three studies were higher [21, 27, 28] and one moderate quality [32]. These studies were too heterogeneous for meta-analysis.

There were no significant differences in PPT comparing SMT against extremity manipulation and against exercise, in a single higher quality study [33].

#### Does PPT change over time after SMT?

Within-group change in PPT after SMT ranged from − 0.11 – 1.0 kg/cm^2^ (− 7.7–38.8%), with a mean increase of 0.31 kg/cm^2^ (9.6%). Considering only those with statistically significant increases from baseline, changes ranged from 0.08–1.0 kg/cm^2^ (2.1–38.8%), with a mean of 0.39 kg/cm^2^ (14.6%).

**Fig. 3:**
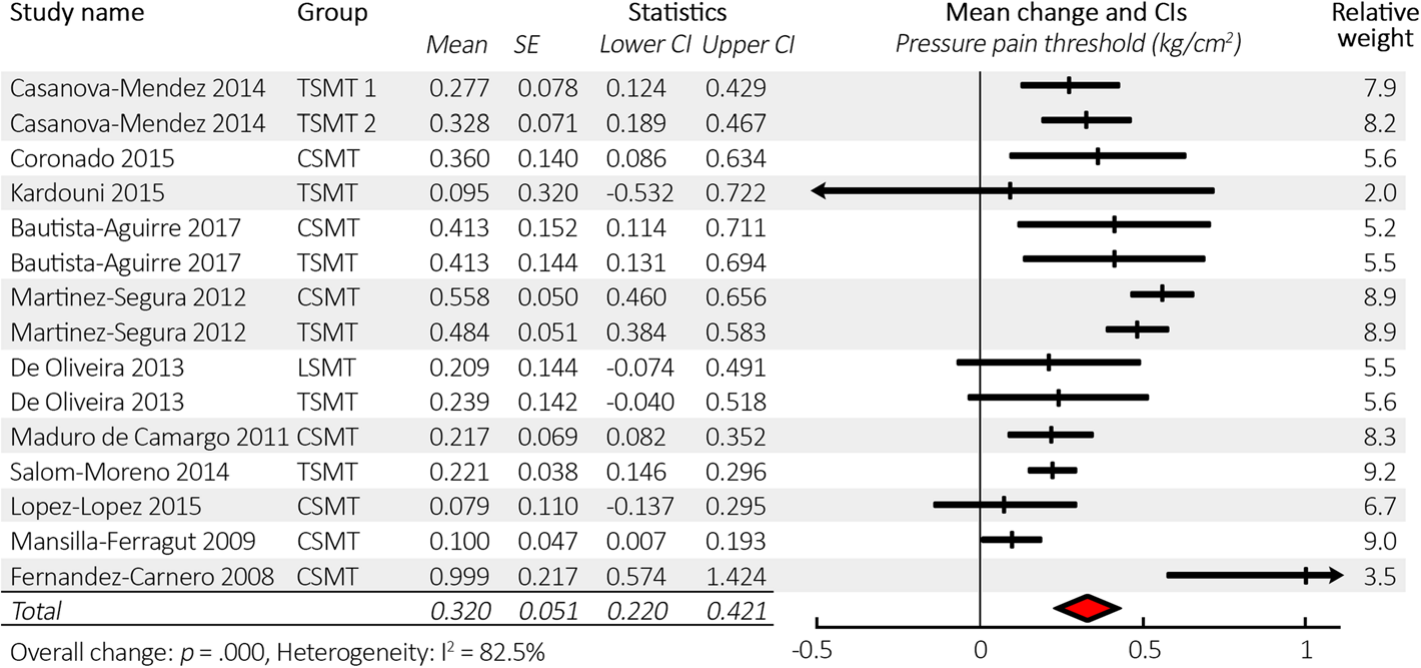
Forest plot of pressure pain threshold change from baseline after spinal manipulative therapy in descending order of study quality. Abbreviations: CI = 95% confidence interval, SE = standard error, SMT = spinal manipulative therapy, CSMT = cervical SMT, TSMT = thoracic SMT, LSMT = lumbosacral SMT. Note: using correlation of 0.75 for calculations of combined variance

Meta-analysis (*N* = 693) (based on combined variances calculating with a correlation of 0.75) revealed that the mean change in PPT from baseline in all SMT groups and all testing locations was 0.32 kg/cm^2^ (CI 0.22–0.42) with *p* < .001. See Fig. 3 for a forest plot, and Table 6 for full results. The mean baseline PPT (factoring in relative weightings as in the meta-analysis) was 2.94 kg/cm^2^, giving a mean increase of 10.9% in PPT over time after SMT.

#### Are any changes in PPT local, regional, or remote?

There were six studies with a total of eight local PPT tests. Four studies observed a local increase in PPT following SMT. These studies were of higher [21, 28], moderate [30], and low [29] quality. Two studies observed no significant change in local PPT following SMT. One study had moderate [32] and one lower [25] quality.

There were nine studies with a total of 13 regional PPT tests. Five studies observed a regional increase in PPT after SMT. These were of higher [21, 33], moderate [30], and lower [26, 34] quality. One study of higher quality observed an increase in PPT at one out of four testing sites [27]. Three studies observed no regional change in PPT after SMT, of higher [35], moderate [32], and lower quality [25].

Eight studies tested PPT at a total of 22 remote sites. Five studies observed a remote increase in PPT after SMT. These were of higher [21, 28, 33] and moderate [31, 32] quality. One higher quality study observed an increase in PPT at one site but not at three others [27]. Two studies did not observe a remote change in PPT after SMT. These studies were higher [35] and moderate [24] quality, one with confirmed and one with attempted blinding. We saw no relation between study quality and result.

**Fig. 4:**
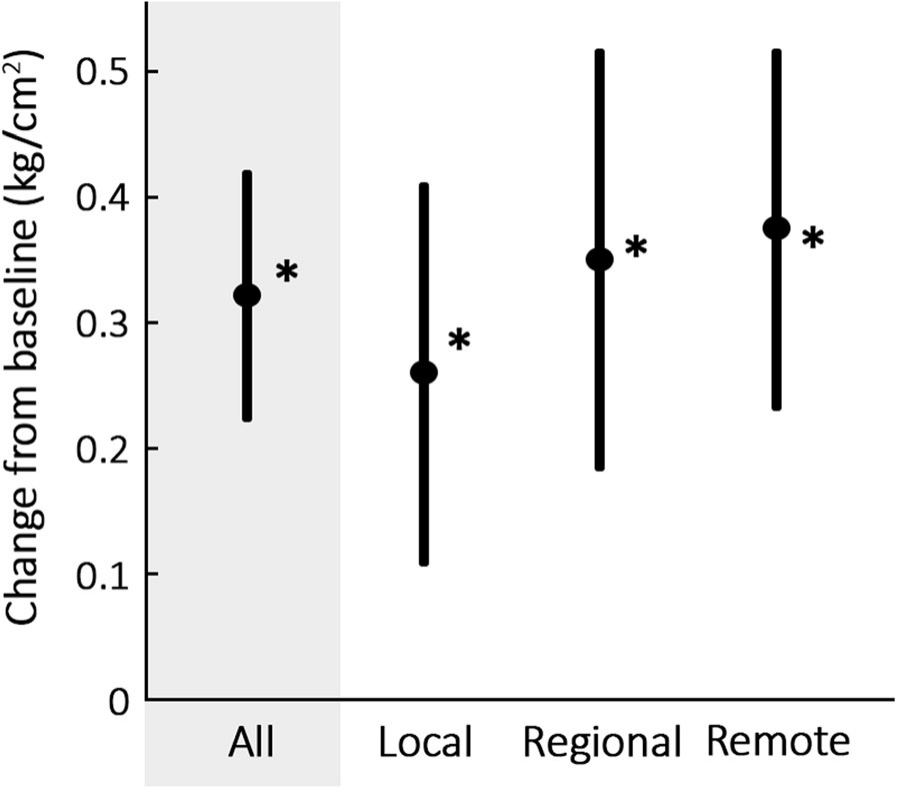
Means and confidence intervals for pressure pain threshold change from baseline after spinal manipulative therapy by testing area. * p ≤ 0.05

Meta-analyses revealed the mean change in PPT from baseline after SMT in local, regional, and remote areas to be 0.26 kg/cm^2^ (CI 0.11–0.41), 0.35 kg/cm^2^ (CI 0.18–0.52), and 0.37 kg/cm^2^ (CI 0.23–0.52) respectively, all with *p* ≤ .001 (based on correlation of 0.75 for combined variance calculation). Five studies could be included in the local subgroup, eight in the regional subgroup, and seven in the remote subgroup, with *N* = 383, *N* = 533, and *N* = 561 respectively. See Fig. 4, and Table 6 for full results.

#### Additional observations

Significant changes in PPT over time were not isolated to any one category of chronicity, and occurred in neck pain and extremity pain populations but inconsistently in low back pain populations. Studies investigating cervical SMT consistently demonstrated a significant increase in PPT over time, which was inconsistent after thoracic SMT and did not occur after lumbar SMT (regardless of musculoskeletal pain site).

Two studies measured PPT at multiple same-day follow-ups. A higher quality study observed that PPT increased from the immediate post-intervention to the 20 min follow-up, in two SMT groups [28]. A lower quality study had no consistent pattern over three short-term follow-ups [25].

#### Other types of quantitative sensory testing

Two studies measured temporal summation, one of which found that temporal summation decreased after lumbosacral SMT over time and compared to two exercise groups, in the lower extremity but not the upper extremity [22]. The other study did not analyse or report the post-intervention temporal summation data [33]. Suprathreshold heat response was measured in a single study [23], observing a significant decrease following lumbosacral SMT compared to sham and control conditions.

Four studies investigated five other types of QST, including heat pain threshold [33, 34], cold pain threshold [34], Aδ “first” pain [22], suprathreshold mechanical pain sensitivity [23], and aftersensations [23]. They all observed no significant change after SMT over time or compared to another intervention. Quality was not assessed in these studies.

## Discussion

### Summary

To our knowledge this is the first systematic review studying the literature on changes in QST measures after HVLA SMT in populations with musculoskeletal pain. Our results indicate that PPT increases systemically over time following SMT in musculoskeletal pain populations in the short term. However, there was no significant difference when compared to sham manipulation. Based on a few studies, there were also no differences between SMT and control or other interventions, which included mobilisation, exercise, and other types of SMT. There were too few studies investigating other types of QST to make robust conclusions.

### Explanation and comparisons

#### Effect of spinal manipulative therapy on pressure pain threshold

There is low quality evidence that SMT does not provide an increase in PPT beyond that observed after a sham manipulation. With a sample size of 92 and only three studies included in the SMT versus sham meta-analysis, it is possible that the meta-analysis is underpowered and at risk of producing a false negative result. We also acknowledge that the sham manipulations could technically be considered as low-grade sustained mobilisations. It is therefore possible that they may elicit a neurophysiological response in their own right, which would confound the results. However, in three of four sham groups there was no increase in PPT after intervention, suggesting minimal placebo effect occurred in these studies. Based on these findings, it is difficult to speculate on whether the significant change over time after SMT observed in the included studies is due to treatment-specific effects or non-specific effects (expectation and contextual factors involved in the delivery of SMT). The systemic nature of the change over time would suggest that a central and systemic hypoalgesic mechanism may be at play, rather than local or regional. It is also important to note that few studies measure beyond 5–10 min post-intervention, hence we could only investigate very short term change in PPT, further limiting the clinical applicability of our results. Heterogeneity in the meta-analyses was high, reflecting the significant between-study variation (in populations, QST locations, and interventions). This suggests that there are real differences in effect sizes between studies.

Our review agrees with prior reviews on the topic of MIH for PPT change over time, and builds upon their conclusions by offering support for the systemic nature of the change [10–12]. Our SMT versus sham results are in contrast to the review by Honoré et al. [13], which concluded in favour of a specific treatment effect in asymptomatic populations. In attempting to compare our meta-analysis results with those of Coronado et al. [10], we noted that their meta-analysis encompassed all between-group differences, with comparators including active, sham, and control interventions. The difference is reported as Hedges’ *g* = 0.32, which is a small effect size. It is difficult to compare against our results, since we consider their meta-analysis inappropriate given that the comparators are highly heterogeneous.

Changes in PPT are most consistently demonstrated after cervical SMT and fairly consistently after thoracic SMT. Both lumbosacral SMT studies showed no change, agreeing with the findings of a review in asymptomatic populations [13]. It is possible that changes over time in PPT do not occur after lumbosacral SMT. The changes over time are not isolated to particular musculoskeletal pain populations, appearing to occur regardless of chronicity and spinal or non-spinal pain site.

#### Clinical relevance of change in pressure pain threshold

The clinical relevance of PPT is an important consideration. It is pertinent to note that statistically significant changes in many short term outcome measures following manual therapy are common and should be interpreted cautiously, since they don’t necessarily relate to clinically important outcomes over meaningful time periods [36]. Articles have stated values for clinically relevant change in PPT of 15% [11] and 1.1 kg/cm^2^ [37], but on inspecting references neither of these are based on the relationship between change in PPT and change in clinically relevant outcome measures. The origin of the 15% value [11] cannot be traced to any provided references, and the 1.1 kg/cm^2^ value [37] was calculated via a distribution-based method for estimating clinically importance difference [38], using effect sizes and standard error of the mean of PPT. There is some evidence, however, that PPT is responsive to change in symptoms, especially to rule in change, based on a study in which change in PPT at the upper trapezius (particularly a change of over 0.86 kg/cm^2^) had high specificity and moderate sensitivity for concurrent change in neck pain over 1 week follow-up [39].

In the absence of a clearly defined and valid minimum clinically important difference, it is valuable to consider the minimum detectable change in PPT, which is the minimum change that would be greater than measurement error or chance. This has been calculated as between about 0.5 and 3.4 kg/cm^2^ (20–50% change) for PPT [19, 39–41]. The change over time results in our review (0.32 kg/cm^2^ or 10.9%) are clearly less than the proposed minimum detectable change. Therefore, we cannot rule out the effect of measurement error and chance on the results.

#### Effect of spinal manipulative therapy on other types of quantitative sensory testing

Our review found single studies for each of temporal summation and suprathreshold heat response that observed a significant reduction following SMT, compared to exercise and sham respectively. The review by Millan et al. [12] comments that temporal summation does not change after SMT. However, on inspecting the three temporal summation studies they included (one of which was also included in our review), temporal summation was reduced after SMT in each study, suggesting a mistake on the authors’ part. Changes in temporal summation after SMT may be worth further study.

Five other types of QST, including thermal pain thresholds, did not change over time or compared to other interventions. Two prior reviews also conclude that there are no changes in thermal pain thresholds [11, 12], based on a total of seven unique studies with some overlap between reviews. Thus it appears likely that thermal pain thresholds do not change after SMT.

### Methodological considerations for this review

We consider it a strength that a comprehensive literature review revealed a large number of studies that fit our criteria, and we were able to perform quantitative analysis to complement the qualitative review. Our quality assessment tool was developed to fit our specific research questions. This may be considered as both a strength, since only relevant items were considered, and also a weakness, since the tool is not standardised. However, there was no standardised tool that fit our needs. We also acknowledge concerns regarding the use of summary scores for assessing study quality, hence we did not exclude studies based on quality but used it as a guide to interpretation.

### Methodological considerations for included studies

Several pertinent quality-related items were addressed poorly in the included studies. Firstly, we have concerns about the sham interventions. Only two studies [24, 35] reported attempting to blind participants, one of which confirmed that blinding was effective [35]. All four sham-controlled studies [24, 26, 34, 35] used a sham that involved holding the participant in a pre-manipulative position, but without a thrust or joint tension. This would account for some, but not all, of the factors proposed by Puhl et al. [42] as important for sham manipulation. They suggest that, in order for a sham manipulation to be convincing, consideration should be given to replicating (or concealing) the physical contact between patient and practitioner, the motions induced during the procedure, the thrust, and the sound (cavitation). Thus it is questionable whether expectation effects were effectively accounted for in three of the four studies. Inadequate control for placebo effects increases the likelihood that results would favour SMT, though we found no significant difference in PPT between SMT and sham in the face of this.

Worryingly, all but one study failed to report on missing data and subsequent imputation methods. Two studies [24, 25] also failed to report adequately on within-group change over time results. Sample size calculations were inadequate in numerous studies; only six had an appropriate sample size calculation based on PPT estimates and met power. We suggest consulting the following article and corrigendum for sample size calculations for PPT [43, 44]. Few studies noted whether study participants were kept naïve to the study aims, which may be valuable in reducing expectancy effects.

A single study controlled statistically for psychosocial factors (e.g. anxiety, depression, pain catastrophizing) [29]. The influence of psychosocial factors on QST measures is disputed; they have been shown to be both relevant [22, 39, 45–47] and irrelevant [22, 39, 47] in various situations. They may be especially pertinent in clinical populations, though randomised controlled trials with QST tend to have poor external validity, thus psychosocial factors may have different importance in these types of trials. We suggest that researchers consider administering psychosocial questionnaires, allowing them to see if statistical control is appropriate based on their data.

We are pleased that all studies utilised assessor blinding and that most studies reported losses and exclusions and between-group differences appropriately.

### Recommendations for future research

There are various limitations to the present studies on MIH, and further studies may shed additional light on the topic. The significant heterogeneity between studies is problematic, as is the lack of quality sham-controlled studies. As Millan et al. [12] suggested, there is a need to focus on more specific research questions in MIH research. We suggest one of the next critical steps is to determine the clinical relevance of MIH. Do changes in QST after SMT relate to clinical features and, more importantly, clinical outcomes for patients? If not, then we can presume that while MIH may represent some specific or non-specific neurophysiologic response, it does not in itself explain the positive clinical outcomes commonly seen after SMT. With these points in mind, Table 7 contains a list of recommendations for future research on MIH.

**Table 7:** List of recommendations for future studies on manipulation-induced hypoalgesia

1.	Use CONSORT guidelines to improve study quality and reporting.
2.	Consider measuring other types of QST apart from PPT, e.g. temporal summation.
3.	Measure QST in a variety of locations, e.g. local, regional, remote.
4.	To address the significant between-study heterogeneity, consider choosing commonly used QST locations, standard QST protocols, and commonly used intervention protocols.
5.	Consult the following article and corrigendum [43, 44] for appropriate sample size estimations for PPT.
6.	Consider including a sham group, and ensure sham interventions are appropriate and believable, and assess the effectiveness of blinding.
7.	Consider comparing changes in QST against clinically relevant baseline features or treatment outcomes.
8.	Consider assessing psychosocial variables at baseline for use as modifiers/confounding variables, if appropriate based on statistical analysis.

## Conclusion

We considered the articles to be generally of low quality. We found systemically increased pressure pain thresholds (reduced sensitivity) over time after SMT of roughly 10% in musculoskeletal pain populations. There was low quality evidence of no difference in PPT after SMT compared to sham manipulation. There were insufficient studies comparing SMT with other interventions and with other types of QST to make further robust conclusions. We make several recommendations for future MIH research. In particular, research into the clinical relevance of MIH, and different types of QST, are likely the most valuable.

## Abbreviations

CI: 95% confidence interval
HVLA: High-velocity low-amplitude
MIH: Manipulation-induced hypoalgesia
PPT: Pressure pain threshold
QST: Quantitative sensory testing
SD: Standard deviation
SMT: Spinal manipulative therapy
